# Efficient Sufficiency: A qualitative evaluation of a 1 year pilot study of young people and parents accessing a mental health drop‐in centre in a paediatric hospital

**DOI:** 10.1111/cch.13051

**Published:** 2022-09-05

**Authors:** Matteo Catanzano, Sophie D. Bennett, Kate Fifield, Laila Xu, Charlotte Sanderson, Anna E. Coughtrey, Ellie Kerry, Holan Liang, Isobel Heyman, Roz Shafran

**Affiliations:** ^1^ UCL Great Ormond Street Institute of Child Health London UK; ^2^ Great Ormond Street Hospital NHS Foundation Trust London UK

**Keywords:** acceptability, cognitive‐behavioural therapy, evidence‐based practice, long‐term condition

## Abstract

**Background:**

Children and young people with long‐term physical health conditions (LTC) are known to have higher levels of co‐morbid mental health problems than medically healthy children. Evidence‐based treatments for mental health problems are effective in children who also have an LTC. This study aimed to explore the factors associated with participants' perceived acceptability and impact of a transdiagnostic mental health centre offering brief psychological assessment and treatment for children and young people and/or their families with mental health needs in the context of long term physical conditions.

**Methods:**

One‐hundred twenty‐eight patients attending the drop‐in centre were invited to participate. Overall, 35 participated (31 parents/carers; 4 children and young people) in semi‐structured interviews (either in person or by phone) exploring their experience of the centre. Interviews were audio‐recorded, transcribed and checked. Framework analysis was then conducted on all transcripts.

**Results:**

Overall, participants found the drop‐in centre highly acceptable and reported a positive experience. Reasons for this varied but broadly focused around four themes: (1) efficient sufficiency; (2) autonomy; (3) fusion of process and content factors and (4) (dis)parities of esteems and ‘seeing both sides of the coin’.

**Conclusions:**

Participants found the intervention acceptable. A mental health drop‐in centre in a paediatric hospital appears to be a positive and valued adjunct to supplement existing mental health services.

## 1 INTRODUCTION

Children and young people with long‐term physical health conditions (LTC) are known to have higher levels of psychiatric co‐morbidity than medically healthy children (Zhou et al., 2019). Psychiatric co‐morbidities are known to negatively impact the young person's quality of life (Baca et al., 2011) and to be expensive for health services (Zima et al., 2016). Whilst evidence‐based treatments for mental health problems in children exist and are effective in those who also have a LTC (Moore et al., 2019), many young people with LTCs are not able to access mental health services (Children's Commissioner, 2016), and those that are may not receive evidence‐based intervention (Welch et al., 2018).

To increase access, brief CBT interventions have been developed based on traditional CBT ‘high‐intensity’ protocols consisting of 12–16 sessions and evaluated in randomized trials. These brief interventions typically comprise either just a single‐session, for example, of psychoeducation, or 6–8 sessions of an intervention, involve technology or other self‐help materials and are delivered by health professionals (Lorentzen et al., 2020; Schleider & Weisz, 2017). Brief psychological interventions have been shown to be effective for common mental health problems in general (Bennett et al., 2019) and in young people with LTCs (Catanzano, Bennett, Sanderson, et al., 2020).

A drop‐in centre in a paediatric hospital delivering brief cognitive behavioural interventions may be one way for more children to access evidence‐based treatments. This was the goal of the ‘Lucy project’: to evaluate the acceptability, feasibility and impact of a ‘Mental Health and Psychological Wellbeing Drop‐in Centre’ in a tertiary paediatric hospital setting (Catanzano et al., 2021) and the quantitative outcomes are reported elsewhere (Bennett et al., 2021; Catanzano, Bennett, Kerry, et al., 2020; Clarke et al., 2022). The project was an uncontrolled trial of young people, their siblings and carers attending a national paediatric hospital. A ‘drop‐in’ booth served both as a focus for recruitment and for raising awareness of the project. Once families had consented and completed baseline measures, an initial triage assessment was carried out by newly qualified clinical psychologists, trained psychological well‐being practitioners (i.e., individuals trained specifically in low‐intensity therapies through a specific programme as part of UK Improving Access to Psychological Therapies initiative) or a junior doctor. All participants were then discussed in a weekly triage meeting with a consultant child and adolescent psychiatrist and allocated to an intervention.

Participants were allocated to (i) provision of/direction to self‐help materials and/or online resources, (ii) further assessment in the form of either a neurodevelopmental assessment and/or computerized mental health diagnostic assessment (the Development and Wellbeing Assessment), (iii) signposting/referral to appropriate internal or external services (including mental health services for adults if the parent had significant symptoms of anxiety and depression) and (iv) a brief modular psychological intervention defined as up to 6 sessions (6 hours total) of either telephone or face‐to‐face (videoconferencing software was not used in this study) guided self‐help. These categories were not mutually exclusive and participants could be allocated to more than one intervention.

As part of the Lucy Project's pilot phase (Catanzano et al., 2021), a qualitative study was conducted aiming to explore the factors associated with participants' (i.e., young people with long‐term conditions and their siblings/parents/carers) perceived acceptability and impact of a transdiagnostic mental health centre offering brief psychological assessment and treatment for children and young people and/or their families with mental health needs in the context of long term physical conditions.

## 2 METHODOLOGY

### 2.1 Design

The design is a cross‐sectional qualitative research study based on semi‐structured interviews.

### 2.2 Ethics

Informed consent was taken for all participants included in the study. Written informed consent was taken for all participants (parents, siblings and index children aged 16 and above who had capacity to consent) included in the study by research assistants. In some instances, participants verbally consented over the phone, which was recorded, and the responses were written up by the research assistants. In the case of children under the age of 16 years, assent was obtained from the relevant child (i.e., sibling, index child or both) alongside parental consent.

### 2.3 Participant selection and sampling

All 128 participants taking part in the pilot phase of a feasibility and acceptability study of a paediatric mental health drop‐in centre between January 2018 and January 2019 (Catanzano et al., 2021) were invited to take part to maximize generalizability. Recruitment, characteristics and a breakdown of the sample from the pilot phase have been described in detail elsewhere (Catanzano et al., 2021).

### 2.4 Setting

All participants in the main pilot study were patients and/or their relatives at a national paediatric hospital.

### 2.5 Study procedure

Participants were invited to a qualitative interview when they were asked to complete the 6‐month study outcome measures. In most cases, this was over the phone, but in some cases, the 6‐month follow‐up was conducted face to face. If they expressed interest, informed consent was then taken, and participants were interviewed either in person or by phone according to their preference. Interviews were semi‐structured and adapted from a schedule devised by the study team from a previous qualitative study (Bennett et al., 2018). This included both key questions and prompts, which interviewers could use to help participants share their experience (Table S1). At the end of each interview, a series of questions were asked about the experience of the interview itself. The interview schedule was piloted with one participant, and no changes were made as a result. This pilot interview is aggregated with the full dataset. In cases where more than one member from the same family was interviewed, we interviewed the parent and child separately (*n* = 4). The interviews were conducted either by phone (*n* = 31) or in some cases face to face (*n* = 4) in clinic rooms at the hospital depending on patient preference. The only people present were the researchers and the participants. Interviews were conducted by MC (male doctor and PhD student, working on the research project as a researcher involved in delivering the intervention) and/or KF (female undergraduate placement student in psychology, working on the research project as a researcher not involved in delivering the intervention). In cases where MC had been the primary therapist for a particular participant, KF would carry out the interview so as to limit bias.

### 2.6 Analysis

All interviews were audio‐recorded, transcribed (anonymizing patient data) and checked. Framework analysis (Gale et al., 2013; Ritchie & Spencer, 1994) was applied to all transcripts using NVivo 12 (QRS International Pty Ltd). This involved the following steps: familiarization, identifying a thematic framework, indexing, charting, mapping and interpretation (Ritchie & Spencer, 1994). We primarily used an inductive (‘bottom‐up’) approach, whereby code development was driven by the data. A priori questions around barriers/facilitators and study aims were also used to develop the initial thematic framework. The two main analysts, MC and KF, met regularly during the process of developing the themes. Any discrepancies or disagreements were discussed with SB. MC's involvement in delivering the intervention conferred some advantages in terms of insight and deeper knowledge of the topic. On the other hand, there are disadvantages to being a part of the intervention being assessed, as it may lead to positive bias; that is, positive comments get focused on, and critical feedback gets minimized. We sought to address this by maintaining curiosity about the data, working closely with members of the research team less involved with delivering the intervention and welcoming a variety of views. MC and LX then re‐applied the final themes to the data to check fit. Inter‐rater reliability was calculated and was found to be strong (kappa = 0.87). The number of participants who endorsed each theme was then calculated. Outcomes are reported in accordance with the Consolidated Criteria for Reporting Qualitative Research (Tong et al., 2007). Finally, descriptive statistics were carried out using SPSS statistical analysis software (version 25, IBM). Participant demographics and symptom profiles were compared to those of the wider sample who were not interviewed. We conducted respondent validation to ensure the researchers' understanding of the interviews was accurate and whether they agreed and/or had anything to add to the summary. All participants were invited to take part in respondent validation. The two participants who responded said they agreed and the themes did not change as a result of respondent validation.

## 3 RESULTS

### 3.1 Sample

**FIGURE 1:**
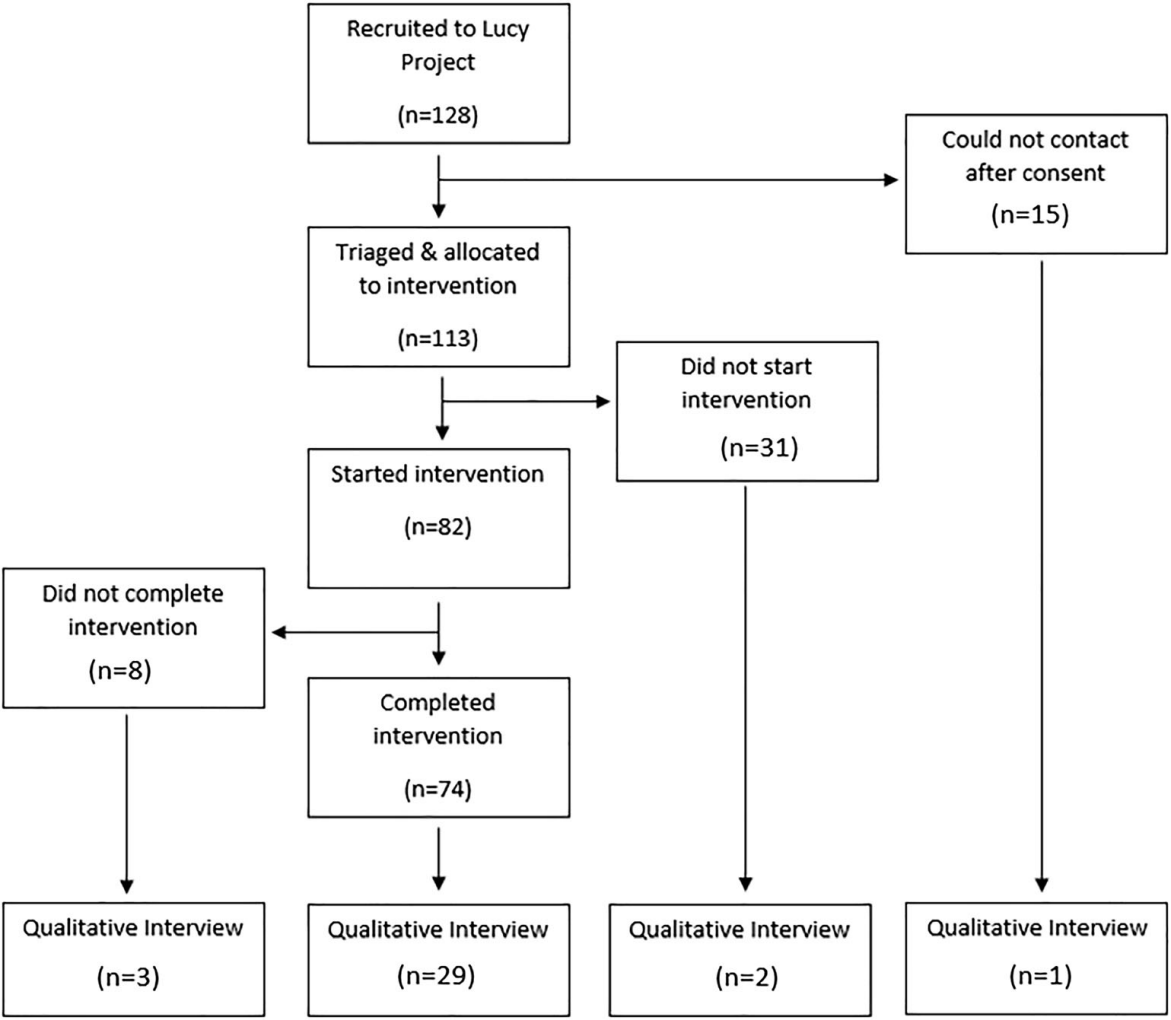
Flowchart of participation in qualitative interviews

Of the 128 who consented to the study, 35 participants from 31 families took part (in the case of 4 families, the child was interviewed in addition to the parent). Four out of 31 parents/carers were fathers, and one out of four young people was a boy. A breakdown of reasons for non‐participation can be seen in Figure 1.

Participants who took part in the qualitative interviews were more likely (49%) to have been allocated to a brief psychological intervention, compared to those who did not (21%) take part (*p* < 0.05). There were no other significant differences between those who participated and those who did not participate in the qualitative interviews (*p* > 0.05). Of those where child mental health data were available at both time points, 15 showed ‘improvement’, 7 ‘deterioration’ and 2 ‘no change’. Scores on the child mental health measure for the subset who took part in the qualitative study are presented in Table S2. Interviews were on average 24 minutes in length (range: 7–47 minutes). Demographic characteristics can be seen in Table 1.

**TABLE 1:** Interview participant demographics

		Participants interviewed(*n* = 35)
Age of index child, median (IQR)		9 (6–12)
Index of multiple deprivation decile, median (IQR)		5 (3–9)
Gender, %	Female	60
	Male	40
Who was the intervention for, %	Patient	80
	Parent/carer	11
	Sibling	9
Ethnicity, %	White	65.5
	Asian	14
	Black	6
	Any mixed background	8.5
	Any other ethnicity	6
Presenting problems, %	Anxiety	40
	Challenging behaviour	34
	Low mood	17
	Other	9
Known pre‐existing neurodevelopmental diagnosis, %	ASD	10
	Intellectual disability (ID)	22.5
	None	67.5
Need for translator, %	Yes	6
History of mental health input, %	Yes	41
History of risk present, %	Yes	9
Primary intervention allocated to, %	Brief psychological intervention	49
	Referral	28.5
	Neurodevelopmental assessment	5.5
	Signposting to resources only	8.5
	Not allocated to intervention	8.5

*Note*: Core participant demographics are shown for all participants who were interviewed along with the median and interquartile range (IQR) and percent (%) of cases (where relevant) for all data. NB: Indices of multiple deprivation (IMD) have been developed in England and Wales to encompass material deprivation and aspects such as health, education and crime. An IMD decile is a dimension that places the deprivation scores of individual areas into one of 10 groups of equal frequency, ranging from the 10% most deprived areas (score of 1) to the 10% least deprived areas (score of 10). These can be used to show the relative deprivation level of the area the participant lives in.

### 3.2 Overview of findings

Overall, participants reported that they experienced the intervention as ‘really helpful’, ‘an amazing resource’ and ‘like it changed their life’. Responses were organized into the following themes: (1) efficient sufficiency; (2) autonomy; (3) fusion of process and content factors and (4) (dis)parities of esteems and ‘seeing both sides of the coin’. The breakdown of themes endorsed by each participant are detailed in Table 2 and by intervention in Table 3.

1. Efficient sufficiency

**TABLE 2:** Themes and subthemes endorsed by each participant and intervention received

Participants	Intervention received	1. Efficient sufficiency	1.1. Rapid access and early intervention	1.2. Low‐intensity and high sufficiency	2. Autonomy	2.1. Flexibility of therapy	2.2. Becoming my own therapist	3. Fusion of process and content factors	3.1. Content factors	3.2. Process factors	4. (Dis)parities of esteems	4.1. Between physical and mental health	4.2. Between the young person and their family
1.1	Referral	x	x	x	x		x	x	x	x	x	x	
1.2	Referral	x	x	x	x	x	x	x	x	x	x	x	
2	Referral				x	x	x	x	x	x	x	x	
3	Brief psychological intervention	x		x	x		x	x	x	x	x	x	
4	Signposting	x	x	x	x	x	x	x	x	x	x	x	x
5.1	Neuropsychiatric assessment	x	x	x				x	x		x	x	
5.2	Neuropsychiatric assessment	x	x	x	x	x	x	x	x	x	x	x	
6	Referral	x	x		x	x		x	x				
7	Brief psychological intervention	x	x	x	x	x	x	x	x	x			
8	Referral	x	x	x				x	x		x	x	
9	Referral	x	x	x	x		x	x	x		x	x	
10	Referral	x	x					x	x	x	x	x	
11.1	Brief psychological intervention	x		x	x		x	x	x	x	x	x	
11.2	Brief psychological intervention	x	x	x	x		x	x	x	x	x	x	x
12	Referral	x	x	x				x	x	x	x	x	x
13	Referral	x	x					x		x	x	x	
14	Signposting	x	x	x	x	x		x	x	x	x	x	
15	Brief psychological intervention	x	x	x	x	x	x	x	x	x	x	x	
16.1	Brief psychological intervention				x	x	x	x	x	x	x	x	
16.2	Brief psychological intervention	x	x	x	x	x	x	x	x		x	x	
17	Brief psychological intervention				x	x	x	x	x	x	x	x	
18	Brief psychological intervention	x	x	x	x	x	x	x	x	x	x	x	
19	Brief psychological intervention	x	x	x	x	x	x	x	x	x	x	x	
20	Brief psychological intervention	x	x	x	x	x	x	x	x		x	x	
21	Brief psychological intervention	x		x	x	x	x	x	x	x	x	x	x
22	Brief psychological intervention	x	x	x	x	x	x	x	x	x	x	x	x
23	Did not start intervention	x	x	x	x	x		x	x	x			
24	Referral	x	x		x		x	x	x	x	x	x	x
25	Brief psychological intervention	x	x	x	x	x	x	x	x	x	x	x	
26	Brief psychological intervention	x	x	x	x	x	x	x	x	x	x	x	x
27	Could not contact after consent before triage	x	x		x		x	x	x	x	x	x	
28	Brief psychological intervention	x	x	x	x	x	x	x	x	x	x	x	x
29	Brief psychological intervention	x	x	x	x	x	x	x	x	x	x	x	x
30	Signposting	x	x		x		x	x	x	x	x	x	
31	Did not start intervention				x	x					x	x	
**Total %**		89	80	71	86	63	74	97	94	80	91	91	26

**TABLE 3:** Themes and subthemes endorsed by intervention type

		Brief psychological intervention%	Other %	Total %
1. Efficient sufficiency		93	86	89
1.1. Rapid access and early intervention	Positive	71	86	80
	Negative	0	24	14
1.2. Low‐intensity and high sufficiency	Positive	79	38	54
	Negative	36	19	26
2. Autonomy		100	76	86
2.1. Flexibility of therapy	Positive	79	48	60
	Negative	43	29	34
2.2. Becoming my own therapist	Positive	100	43	66
	Negative	21	14	17
3. Fusion of process and content factors		100	95	97
3.1. Content factors	Positive	100	86	91
	Negative	21	67	49
3.2. Process factors	Positive	79	76	77
	Negative	29	10	17
4. (Dis)parities of esteems		100	86	91
4.1. Between physical and mental health		100	86	91
4.1.1. Impact of intervention on physical and mental health	Affected physical health	43	38	40
	Did not affect physical health	36	24	29
4.1.2. Integration of physical and mental health		36	43	40
4.1.3. Therapists as LTC experts	Important	64	57	60
	Not important	29	33	31
4.2. Between the young person and their family		43	14	26

The first theme relates to the tension between increasing access and giving participants a sufficient ‘dose’ of an intervention. Overall, participants reported finding the intervention sufficient and highly efficient. Factors such as being referred, but not being seen by the time the interview was conducted, explained the minority of cases who would have liked to receive ‘more’.

1.1. Rapid access and early intervention

Parents and young people (28/35; 80%) were relieved they were able to access support quickly, especially because of high thresholds in local CAMHS:

I'd say I think it's really important and I'd say by being one of the lucky people who got help quickly it's made a real difference and I think people should be able to go to someone and within kind of a month or you know or a short period of time. (Referral, PID 1.1 ‐ Child)

Then as she got a bit older it's just been she does not meet the criteria for receiving support. Unfortunately in our local area you have to have tried to commit suicide before they can see you. (Referral, PID 4 ‐ Parent)

Even in cases where the problem was comparatively mild, participants talked about the importance of intervening early to prevent things getting worst or future problems arising:

Even though not now she's not being bullied because she's got a nice set of friends but if we do not deal with this now when she turns 12/13 of course she's going to be bullied and of course they are going take the micky out of her and of course she'll feel unhappy that she cannot go out or go shopping with her friends, which is why I thought it was really important to get the help in that point in time. (Brief psychological intervention, PID 18 – Parent)

1.2. Low‐intensity and high sufficiency

The brief intervention delivered as part of the booth appeared to be acceptable to participants. This seemed to be in part because the improvements in a single behaviour would generalize and positively influence other areas of life:

So I think that this whole project is amazingly beneficial to parents like ourselves that are battling with the system. If for lots of other reasons, and trying to lead a normal life, and hold down jobs [...] just getting that 6–8 weeks of intervention has been hugely beneficial […] you target one thing and the positive behaviour moves into other areas if that makes sense. (Brief psychological intervention, PID 28 ‐ Parent)

The young people themselves reported improvements in understanding of their own mental health:

I thought that it really helped me learn a bit more about OCD and sort of how I cannot struggle with it anymore. (Brief psychological intervention, PID 16.1 – Child)

Participants were really pleased with how quickly they could see changes in other aspects, such as their relationship with other family members (e.g., their child):

Now in 6 weeks we made a relationship. The way I talk to him, you know talking, praising him. (Brief psychological intervention, PID 22 – Parent)

Even in cases where there were still challenging days, the improvement was sufficient to make a considerable difference to the family:

Before I spoke to the people at the Lucy project, her behaviour was getting really unruly, like she would not listen to me at all, through using the tips that I was given, she's now still having a few days here and there when she's not well behaved, but it's nowhere near as bad. (Brief psychological intervention, PID 26 ‐ Parent)

Generally, participants felt like the number of sessions offered was enough and sufficient in addressing their issues, although one participant did mention wanting a follow‐up session:

After that initial 6–8 weeks, what we might find useful is possibly a drop‐in after about a year, to cycle back to see if any of those things aren't working. (Brief psychological intervention, PID 28 ‐ Parent)

2. Autonomy

The second theme focused on the idea of the intervention being flexible in that participants had the ability to make their own choices and adjustments so that the interventions could be tailored around their lives; for example, they liked being able to receive the intervention by phone or face to face. Participants explained that they learnt how to become their own therapists and that this increased their freedom and ability to make choices in their lives; for example, young people described being able to go to the cinema and see friends following treatment for anxiety.

2.1. Flexibility of therapy

Participants (21/35; 60%) really appreciated the flexibility of the intervention. This was true in terms of scheduling appointments around existing commitments, but also the mode of delivery (e.g., phone or face‐to‐face):

There was a real appreciation for how busy, how stressful our lives are in just dealing with everybody and trying to get appointments and coordinate that and the education side as well […] and, [the therapist] would allow us to have meetings if we were coming up for heart appointments. (Brief psychological intervention, PID 28 – Parent)

Although telephone therapy made it more easily accessible, there were still some challenges such as the telephone being a barrier to effective communication and feeling restricted with the timings available:

I think sometimes over‐the‐phone could be a little bit trickier because sometimes the emotions come out when you are in‐front of the person, rather than on the other end of the phone. I think that is probably more a technology thing, which can cause a bit of a barrier. (Brief psychological intervention, PID 16.2 – Parent)

Some participants really appreciated the flexibility in pace of delivery and feeling that was collaboratively decided:

It wasn't forced, so she felt she could do everything at her own speed. (Brief psychological intervention, PID 18 ‐ Parent)

2.2. I became my own therapist

A number of participants (23/35; 66%) commented on being able to internalize the strategies and quickly become ‘their own therapist’:

It was just amazing to the point where I was becoming my own little therapist. I was calling my friends telling them ‘you should do this with your children!’ you know? (Brief psychological intervention, PID 22 ‐ Parent)

Participants liked the autonomy of being able to select the strategies that worked for them:

I mean some of it was useful, some of it did not really apply, but I just used the bits and bobs that I felt would fit in. (Brief psychological intervention, PID 18 ‐ Parent)

A positive aspect in participants becoming their own therapist was that once the strategies had been learned, participants no longer needed the therapist and felt able to continue making progress themselves:

Then that meeting we had with [the therapist], X was pretty much there but by that point, I said to [the therapist] I think we are happy to call it a day because I think from here we can only go from strength to strength because I can see its just practice now. (Brief psychological intervention, PID 18 ‐ Parent)

Following the intervention participants reported feeling able to do things, they previously had not been able to do due to their or their child's mental health problem:

Yeah her reward was ‘mummy I want to go to the cinema with my friends where I do not want to ask their parents to take me on the lift because it's embarrassing’ so that was her best reward to be able to do that and she did! (Brief psychological intervention, PID 18 ‐ Parent)

Yeah, and this is personal circumstance but, I had the opportunity to go for a promotion at work, and actually […] so before we had been on this, we would not have dreamt of it because it would have required a bit more resilience from me and potentially more time in terms of work focus. To then be in a situation where you could see things were making a difference, we were functioning better as parents […] even as far as our relationship has improved – we go out now. (Brief psychological intervention, PID 28 – Parent)

3. Fusion of process and content factors

The third theme highlighted the synergy between process and content factors of the intervention. Families valued both process and content factors. This synergy was best embodied by the therapists who were able to simultaneously be compassionate and professional, but also a source of practical support, with concrete strategies, goals and outcomes.

3.1. Content factors

The large majority of participants (32/35; 91%) appreciated the content of the practical resources (e.g., handouts detailing the strategies) that were given to them by the therapist and these were felt to be highly acceptable:

The handouts we were getting, the video support, were really good. (Brief psychological intervention, PID 28 ‐ Parent)

Beyond the handouts, participants really valued the practical ways in which the therapists guided them through the intervention, by, for example, supporting them to implement the strategies in the handout:

I used to bombard her with all my problems […] I would not be able to see the wood from the trees and she was carving it up and make me step back and answer my questions. (Brief psychological intervention, PID 28 ‐ Parent)

Young people and parents found tracking their progress using goal‐based outcomes helpful and reported a sense of achievement when they could see improvements:

The questionnaires I think that they should keep doing them as well because they really sort of help you look at them and look back and sort of say well I'm a 1 then but now I've made some progress to a 6. […] It makes me feel really happy and it makes me feel that they helped. (Brief psychological intervention, PID 16.1 ‐ Child)

It was amazing how I was moving up numbers every week […] for the six weeks I moved from I think two or three to eight and nine. To me that's a big achievement in six weeks. You know, it something that I could not do […] you know, I've read books, I've watched YouTube videos ‘how to be a good parent’, and it never worked. (Brief psychological intervention, PID 22 ‐ Parent)

Even for participants who were not suitable or did not want the brief psychological intervention via the project, receiving other interventions, such as a referral and accessing services they had previously been unable to access, was found to be highly acceptable:

Without your support we would not have got contact with CAMHS. (Referral, PID 8 ‐ Parent)

For a small number of participants (3/35; 9%), referral/signposting to other services alone, whilst acceptable, was not enough, with participants wanting more practical solutions:

In terms of more practical, immediate things that we could do, that would've been really useful […] to give some strategies for the parents, rather than waiting for an assessment and things like that. (Signposting, PID 14 ‐ Parent)

3.2. Process factors

Aside from the very practical ways in which families benefitted and were affected by the content of the intervention, a number of process factors and benefits were also reported by the majority of participants (27/35; 77%). For example, they discussed feeling believed, relief, reassured, understood, calmer, heard, confident, grateful and hopeful:

We were in a real down problem state and not really sure what to do and what the future will hold but now there is sort of a light at the end of the tunnel, we are getting there and just having someone who listens and understands was great. (Brief psychological intervention, PID 21 – Parent)

Many of these effects were reportedly related to therapist characteristics, which acted as a source of motivation and validation of their difficulties. Therapists were described as empathic, understanding, collaborative, good communicators, having a non‐judgemental attitude, comforting, supportive, natural, normalizing of patient experience, thorough and professional.

All I can say that it was super helpful […] she was so comprehensive, she was a very understanding person and she gave us really, really good advice. (Brief psychological intervention, PID 29 – Parent)

Actively reaching out to families and handing out leaflets helped families overcome mental health stigma and associated feelings of pride and embarrassment in taking that first step and asking for help:

Drop‐in is a bit different because you have to actually put your pride and your embarrassment to the side to actually take yourself there, […] but it was helpful for me because one of you actually came to me. (Referral, PID 2 ‐ Parent)

However, some participants did mention that in cases where the child was an inpatient, parents would not feel able to remove themselves and go to a booth, even if it was in the hospital:

When a parent is in that situation yes they feel all these feelings but me personally I would not take myself out of my son's situation or remove myself from being next to him or near him to go and speak to somebody. (Referral, PID 2 ‐ Parent)

4. (Dis)parities of esteems and ‘seeing both sides of the coin’

The fourth and final theme explored the disconnect between how health and other services separate physical/mental and child/family and how this contrasts both (i) how participants see themselves and their problems (i.e., physical and mental health problems both affecting each other; their whole family being affected by the illness/not just their child) and (ii) participants' desire for better integration.

4.1. Physical and mental health

Integration of physical and mental health care was deemed to be important for acceptability by most (32/35; 91%), in part through co‐location of care and partly because participants did not see mental and physical health as different. For one participant, they were just two facets of their child's life:

Sorry I am very passionate about it, it's just I see both sides of the coin and you just need that support so badly because you are just so stuck. You really are […] there will always be a medication in the cupboard, there will always be a doctor to call but when it comes to mental and emotional you need people around you and you need a team to support you so that's why it's so important. (Brief psychological intervention, PID 17 – Parent)

Most participants (14/35; 40%) reported no change in their own or their child's physical health condition as a result of the intervention:

I do not think changes in her [physical] condition happened because of the input. (Referral, PID 4 – Parent)

Others (10/35; 28%) felt their child's physical condition improved:

So X had really bad psoriasis, she had suffered for about 6 months which I think was a combination of anxiety and when her cousin had her accident, she got it really bad again, but now apart from a tiny patch on her arm, physically its gone. (Brief psychological intervention, PID 11.2 ‐ Parent)

There were a broad range of opinions on the importance of therapists as ‘LTC experts’ ranging from a preference for them having no existing knowledge to it being crucial for effective treatment:

I think she kind of knew what it [the endocrine condition] was. Yeah knew of it rather than know about it […] yeah it was nice for her not to know because she just kind of treated you like you were a normal person seeking help as supposed to someone who had a condition who may affect it so yeah it was nice for her not knowing.

(Referral, PID 1.1 ‐ Child)

I want to speak to someone who knows and has spoken to parents who have been in similar situations to myself, because I do not want to go and speak to a therapist for the sake of speaking to a therapist, I want to speak to somebody that understands, maybe been in a similar situation themselves or have dealt with parents who are in similar situations. (Referral, PID 2 ‐ Parent)

Around a third (11/35; 31%) thought specific knowledge was not required:

No, once you do your ABCs you can, well I am not trained so I [laughter], you can see the links, you can see what the trigger is actually here. So, I do not think, if the board or whoever you have to go to, to get funding for this, say you cannot do this because you are not medical experts and do not know what the different syndromes you are dealing with, I think is nonsense. (Brief psychological intervention, PID 28 ‐ Parent)

And slightly over half (21/35; 60%) thought a general awareness of what it is like to live with a physical health condition or have a child with one, was important, though specific medical knowledge was not:

As long as they are sort of aware of what that will mean to the child, I do not think they need to know the ins and outs of every single illness or diagnosis. (Could not contact after consent and before triage, PID 27 ‐ Parent)

4.2. The young person and their family

Integrating not only physical and mental health but also the young person and their families within the intervention was deemed an important aspect of acceptability. Parents both appreciated being part of the intervention but also reported direct improvement of their own mental health when their child's mental health improved:

There's certainly no other support or help that's out there that allows […] that focuses on the parents or that looks at the parents and says how do you play into this anything other than logistically of just sorting out their assessments and therapies which is a lot of the support. (Brief psychological intervention, PID 28 – Parent)

Because we are a lot less stressed, because I'm not going to pick her up from school and wondering whether she's done something naughty. We're all getting more sleep, we are able to do a lot more together now. (Brief psychological intervention, PID 26 ‐ Parent)

## 4 DISCUSSION

### 4.1 Summary of findings

The main aim of this study was to qualitatively explore the factors associated with participants' perceived acceptability and impact of the intervention. All participants had taken part in the pilot phase of an uncontrolled open trial that showed an increase in quality of life and decrease in emotional and behavioural symptoms (as reported by parents) after attending the drop‐in centre (Catanzano, Bennett, Kerry, et al., 2020). To the authors' knowledge, this is the first qualitative study to explore participants' experiences of a mental health drop‐in centre in a paediatric hospital for young people with long term conditions and their families.

### 4.2 Acceptability

Overall, participants' experience of rapidly accessing a brief intervention was positive. This was especially the case in those who received a brief psychological intervention (e.g., guided self‐help). Conceptually, similar themes to the idea of ‘efficient sufficiency’, under the heading of ‘accessibility’, were noted in a meta‐synthesis of psychological interventions in children and young people with long term conditions by Moore and colleagues, where young people expressed the importance of accessing interventions ‘at the right time’ (Moore et al., 2019). One participant reported that a follow‐up/booster session at 1 year may have been useful: “to cycle back to see if any of those things aren't working.” At present the evidence for the effectiveness of booster sessions at improving outcomes in youth interventions for internalizing and externalizing symptoms is mixed, with more recent evidence failing to find an association between the presence of booster sessions and improved outcomes (Buzasi et al., 2022; Gearing et al., 2013; Sun et al., 2019; van Aar et al., 2017). Van Aar et al. (2017) have suggested there may be subgroups for which booster sessions may be particularly beneficial. Future research should aim to identify these subgroups and test whether offering targeted booster sessions improves outcomes, as well as answer questions of cost‐effectiveness, mechanisms of action and acceptability. The focus on clearly defined goals and witnessing that improvement generalized to other areas contributed to acceptability. The flexibility of delivery inherent in the intervention, by for example offering telephone therapy/scheduling appointments around other medical appointments, appeared to be an important factor for acceptability. Participants felt it was important for the support to include both content (i.e., the self‐help materials and the goals they had achieved) and process factors (feeling validated, reassured, listened to, etc.). Having a therapist who was able to give the participant perspective, help them ‘problem‐solve’ and implement the strategies, whilst simultaneously being empathic and giving patients validation and a sense of hope was one way in which acceptability was increased as parents could get help if they were stuck and did not have to struggle on their own. In the aforementioned meta‐synthesis, conceptually similar themes centring on the ‘therapeutic foundation’ (containing: ‘therapeutic relationship’, ‘safe space’, ‘boundaries’ and ‘unconstrained’) reflected some of the findings of this study surrounding the importance of both process and content factors. Of note, the intervention was primarily delivered over the telephone, yet this did not appear to interfere with the ability to establish a good therapeutic relationship, which supports previous research in remote therapy (Irvine et al., 2020). Though a range of opinions and experiences were shared by participants, the majority reported that although it was helpful that therapists understood what it was like to have a LTC, in‐depth medical knowledge was not deemed necessary. Previous qualitative studies have reported similar findings in children with neurological conditions including epilepsy (Bennett et al., 2015; Jones et al., 2022), diabetes, bronchiectasis unrelated to cystic fibrosis, and epidermolysis bullosa (Jones et al., 2022). The high levels of acceptability described in the study overall may in part be explained by a high degree of overlap between the intervention model (e.g., co‐location, integrated care, self‐referral and facilitating onward referral to local services) and the ways families would like tertiary paediatric outpatient clinics to facilitate access to mental healthcare (Jones et al., 2022).

### 4.3 Impact

Participants reported having choices they did not have before, by for example being able to go for a promotion at work, once the strategies for managing challenging behaviour had been learned. The perceived increase in autonomy (i.e., families feeling more ‘free’, ‘in control’ and with more ‘choices’) may be one way participants were impacted by the intervention. Previous research highlighted in the review by Moore et al. (2019) suggests that empowerment, self‐esteem and self‐management, may feed into each other to affect a young person's resilience and be influenced through psychological intervention, especially when these include learning skills. This resonates with findings in this study, whereby participants felt that once the strategies had been learnt, they felt able to continue managing difficulties on their own. This may be a particular advantage of guided self‐help and CBT interventions, which emphasize learning strategies. Most participants did not report changes to their physical health because of the intervention, although there were some notable exceptions (e.g., a young person with psoriasis). This is in line with quantitative data (Catanzano, Bennett, Kerry, et al., 2020).

### 4.4 Limitations

Study limitations mean the results should be interpreted cautiously. All participants were invited to interview, but in practice, the majority who accepted were participants who had completed a brief intervention (29/35). It is possible that this led to a degree of selection bias, where for those who consented to be interviewed, improvements from the intervention motivated them to take part and ‘give back to the project’ by taking part in the qualitative study at follow‐up. This was counterbalanced by attempting to recruit participants who had not completed the intervention (see Figure 1), and although numbers were smaller (*n* = 6), they still consisted 17% of the sample. Only a minority of participants interviewed had not started the intervention (*n* = 3), making comparisons with the rest of sample limited. Overall, there appeared to be some similarities in terms of participants' recognition that integrated care and accessibility were important (even when they did not receive an intervention themselves). The majority of participants interviewed were mothers (27/35), limiting the extent to which our findings can be applied to fathers and young people. As discussed in more detail elsewhere (Bennett et al., 2021; Catanzano, Bennett, Kerry, et al., 2020; Clarke et al., 2022), the lack of control group is a limitation of the wider study, as improvements may have been due to time or other confounders. We did not use validated measures of service use, which would have captured use of all interventions/support outside the project, as it was felt that questionnaire burden was already high.

### 4.5 Implications for future research

These findings, in conjunction with analysis of quantitative outcomes (Bennett et al., 2021; Catanzano, Bennett, Kerry, et al., 2020; Clarke et al., 2022) and the acceptability and feasibility of remotely delivered training (Batchelor et al., 2020), may be used to help guide the design and implementation of future randomized controlled trials evaluating the effectiveness of brief psychological treatments for young people with LTC. This may be done by, for instance, retaining a degree of flexibility in terms of scheduling appointments, using goal‐based outcomes and routine outcome monitoring as part of the intervention and offering brief CBT/guided self‐help.

## CONFLICT OF INTEREST

The authors declare no conflict of interest.

## ETHICS STATEMENT

Ethics approval was granted by the London Riverside Research Ethics Committee (REC reference number: 16/LO/1915).

## Supporting information


**Table S1.** Interview schedule: example questions and associated prompts.
**Table S2.** Medians and interquartile ranges (IQR) of the Strengths and Difficulties Questionnaire (SDQ) scores of those who completed a qualitative interview.

## Data Availability

The anonymized data that support the findings of this study are available from the corresponding author upon reasonable request.
