# Supplementary material for: Efficient Sufficiency: A qualitative evaluation of a 1 year pilot study of young people and parents accessing a mental health drop‐in centre in a paediatric hospital
Source: Child Care Health Dev. 2022 Sep 5;49(2):332–45. doi: 10.1111/cch.13051 (PMC10087919; doi:10.1111/cch.13051)
Supplement: Supplementary file 1 — Table S1. Interview schedule: example questions and associated prompts. Table S2. Medians and interquartile ranges (IQR) of the Strengths and Difficulties Questionnaire (SDQ) scores of those who completed a qualitative interview. [file CCH-49-332-s001.docx]

**Table 1S.** Interview schedule: example questions and associated prompts.

| Stem | Probes |
| --- | --- |
| Overall, what was your  experience of taking part in the  drop-in centre study? | How easy would you say it has -or has not- been to contact the drop-in centre?  Are there any ways that you think that we could improve your experience of accessing the Lucy project / drop-in centre? |
| What was your experience of  completing the questionnaires? | What was good/bad  Is it acceptable? |
| What are your views about the  initial assessment you had? | Needed?  Hassle?  Relevant/non – relevant questions?  What did you like/dislike about doing the assessment?  (If done over the phone) would it have been better to do it face-to-face? |
| What was your experience of the support you were offered? | What was good/bad?  Useful/not useful? – if so which parts were most useful – least useful  Is it acceptable?  Do you think it will be useful in the future?  If you did it f2f how was that? Would you have preferred to do it over the phone? (or reverse)  If you had a mix – which one did you prefer? Was it good/bad having a mix? |
| Before the Lucy Booth, had you tried seeking support from CAMHS? From your GP? From psychological services at the hospital? | How easy was this?  How helpful was this?  Why did you come to the Lucy booth if you were seeking/having help elsewhere? |
| If yes and successful – How would you compare the treatment received during the study to past treatments? | How was this similar?  How did it differ?  Which did you prefer and why? |
| Did you see any changes in you or your child’s mental health as a result of the treatment? | If so, how?  If not, why do you think?  Before this, did you think that X’s emotional and behavioural needs were something that could be addressed with psychological treatment or did you just see them as part of the medical condition?  What was the experience of the rest of the family? |
| Did you see any changes in your  child’s medical condition as a result of changes  in mental health? | What type of changes?  Why do you think these changes happened?  Did you notice any changes in the appointments with your child’s medical consultant because you/X were receiving mental health support elsewhere. |
| How was your therapist knowledge of X’s medical condition? | How important was this for you? |
| What would you tell  other parents considering taking  part? | Any Advice?  What to do or what not to do? |
| What would you say to the people deciding whether to fund this as a service? | Reasons why? |
| Finishing the interview:  I’d like to finish by asking you  some questions about your  experiences of taking part in the  interview today.  Is there anything that we haven’t  talked about that is important?  Are there any questions we  should add/change/remove?  What has it been like doing the  interview over the phone? What  would make it easier/better?  Have you found the length of the  interview acceptable? |  |

**Table 2S.** Medians and interquartile ranges (IQR) of the Strengths and Difficulties Questionnaire (SDQ) scores of those who completed a qualitative interview.

| Measure | Pre | |  | Post | |
| --- | --- | --- | --- | --- | --- |
|  | n | Median (IQR) |  | n | Median (IQR) |
| SDQ Total Score | 24 | 18 (12-25) |  | 24 | 17.50 (10-25) |
| Impact | 23 | 4 (2-6) |  | 17 | 5 (1-8) |
| Emotional | 24 | 5 (3-9) |  | 24 | 4.50 (3-7) |
| Conduct | 24 | 3.50 (1-6) |  | 24 | 3 (1-5) |
| Hyperactivity | 24 | 6.50 (4-9) |  | 24 | 6 (4-9) |
| Peer | 24 | 4 (1-5) |  | 24 | 3 (1-6) |
| Prosocial | 24 | 7 (4-8) |  | 17 | 7 (6-10) |
